# The Research Progress on the Interaction between Mammalian Gut Microbiota and the Host’s Metabolism Homeostasis during Hibernation

**DOI:** 10.3390/metabo14030134

**Published:** 2024-02-21

**Authors:** Zhepei Zhang, Fengcheng Song, Linjuan Wang, Zhengrong Yuan

**Affiliations:** College of Biological Sciences and Technology, Beijing Forestry University, Beijing 100083, China; zzp1011@bjfu.edu.cn (Z.Z.); nkjmkksfc218@bjfu.edu.cn (F.S.); wanglinjuan@bjfu.edu.cn (L.W.)

**Keywords:** mammals, metabolism, hibernation, gut microbiota, homeostasis

## Abstract

Hibernating mammals confront seasonal and harsh environmental shifts, prompting a cycle of pre-hibernation feeding and subsequent winter fasting. These adaptive practices induce diverse physiological adjustments within the animal’s body. With the gut microbiota’s metabolic activity being heavily reliant on the host’s diet, this cycle’s primary impact is on this microbial community. When the structure and composition of the gut microbiota changes, corresponding alterations in the interactions occur between these microorganisms and their host. These successive adaptations significantly contribute to the host’s capacity to sustain relatively stable metabolic and immune functions in severe environmental conditions. A thorough investigation into the reciprocal interplay between the host and gut microbiota during hibernation-induced adaptive changes holds promise for unveiling new insights. Understanding the underlying mechanisms driving these interactions may potentially unlock innovative approaches to address extreme pathological conditions in humans.

## 1. Introduction

Hibernation is a physiological strategy employed by certain mammals to cope with harsh environmental conditions and limited food resources during the winter months. During hibernation, animals undergo adaptive changes such as reduced body temperature, slowed metabolism, and decreased heart rate [1]. The dietary composition of hibernating animals exhibits seasonal variations, with most of them increasing their food intake during the summer and the autumn to store fat, while fasting during hibernation [2,3].

The gut microbiota establishes its presence within the host’s gut from birth. This coexistence gives rise to a symbiotic relationship that plays a crucial role in shaping the host’s overall health [4]. They contribute to the development and maintenance of the immune system, aiding in protection against pathogenic microorganisms by complex interactions with the host [5]. Additionally, it plays a role in the synthesis of bioactive compounds that can influence various bodily functions [6]. The gut microbiota also participates in maintaining the integrity of the intestinal barrier, while aiding in regulating the host’s responses to inflammation and stress [7].

Gut microbiota is highly reliant on the energy obtained from the host’s diet to perform vital metabolic functions necessary for their survival [5]. Consequently, the composition of gut microbiota can rapidly respond to changes in dietary habits [8]. During hibernation, alterations in the host’s food composition and shifts in the surrounding environment can significantly impact gut microbiota. Concurrently, these changes can have profound effects on the physiology of hibernating host animals [9]. Current research suggests that the interaction between the host and the gut microbiota during hibernation may be a critical factor, enabling hibernating animals to maintain homeostasis [10,11,12]. Understanding the mechanisms of adaptation between hibernating animals and their gut microbiota during the winter fasting period may contribute to the in-depth exploration of novel therapeutic approaches for metabolic disorders in non-hibernating species [6].

## 2. Effects of Hibernation on the Gut Microbes of Mammalian Animals

### 2.1. The Effects of Hibernation on the Host’s Gut Microbiota Diversity

The diversity of gut microbiota involves the richness of species and the distribution of relative abundance of microorganisms [4]. Different types of bacteria possess different abilities to break down and absorb substrates, leading to their diverse metabolites. Thus, the significance of diversity in the gut microbiota stems from its influence on host health and overall biological balance. The dysregulation of the gut microbiota has the potential to result in a range of metabolic disorders [13,14,15].

Hibernating species like the 13-lined ground squirrel, Daurian ground squirrel, greater horseshoe bat, and brown bear have undergone thorough studies concerning their gut microbiota [2,16,17,18]. Due to winter fasting, the host experiences a sharp reduction in degradable substrates available for gut microbiota, which can impact the variety and composition of the gut microbial community [19]. Previous research has suggested that results from the 16S rRNA gene sequencing of DNA extracted from cecal contents of 13-lined ground squirrel [20] can support the fact that community diversity changes in the hibernator gut microbiota. During hibernation, the Shannon index and Simpson index were significantly decreased compared to other periods, indicating a noticeable reduction in alpha diversity compared to the active phases [20]. In the summer, the predominant phyla in the ground squirrel’s gut microbiota were Bacteroidetes, Firmicutes, and Verrucomicrobia, with the latter being primarily represented by *Akkermansia muciniphila* (*A. muciniphila*) [6]. The relative abundance of Firmicutes, including Lactobacillaceae and classes Lachnospiracea, reduced during hibernation, displaying an almost total absence of Operational Taxonomic Units (OTUs) categorized to obtain taxa, and the same applies to the phylum Mollicutes [20]. In general, the diversity of gut microbiota of 13-lined ground squirrels significantly decreases during hibernation [6,20]. In the study on Daurian ground squirrels, they were divided into three groups: the pre-fattening period, hibernation period, and active period. In terms of alpha diversity, the Shannon index of the fattening and hibernation phases was significantly lower than that of the active phase [21]. The results of the biological analysis indicated that during the pre-fattening period, as Daurian ground squirrels continued to consume food, the dominant microbial communities in their cecum tended to stabilize, which was accompanied by a decrease in the overall microbial abundance. Conversely, the hibernation period was characterized by fasting, which led to a scarcity of nutrients required by the gut microbes, resulting in a reduction in the abundance [21]. Carey et al. found that in the emergence period, as the 13-lined ground squirrels increased their food intake and had access to a more diverse food source, the abundance of gut microbiota reached its highest level [22]. Moreover, in the investigation of hibernating brown bears, a reduction in gut microbiota diversity has been observed [17]. Stool samples were collected from brown bears during both the hibernation and active periods of the same year. Through 16S rRNA analysis and sequencing, the findings indicated a noteworthy reduction in gut microbiota diversity during hibernation as opposed to the active period. The predominant bacterial phyla with elevated relative abundance during hibernation encompassed Proteobacteria, Firmicutes, Bacteroidetes, and Actinobacteria [17]. By utilizing DNA extraction and 16S rRNA sequencing technology, 1750 OTUs resulted from the gut microbiota of 39 bats. By analyzing the alpha diversity, the diversity within the gut microbiota was significantly greater in late summer as opposed to that in early winter. Moreover, the Shannon index of the gut microbiota during early summer exhibited a notably lower value compared to the winter period [18].

Due to the fasting behavior during hibernation, the scarcity of dietary substrates leads to a reduction or even disappearance of certain microbial groups that cannot adapt to extreme conditions [9]. Only a small fraction of these groups possess the ability to switch metabolic substrates or metabolize host-derived substrates during hibernation [23], enabling them to survive in this period. Overall, the diversity of the mammalian gut microbiota during hibernation is significantly decreased compared to that during the active periods. During hibernation, the increase or decrease in specific microbial populations in the host’s gut collectively contributes to a reduction in gut microbiota diversity. This alteration might assume a pivotal role in helping the host adapt to the scarcity of food and fluctuations of the environment during the winter, maintaining relative homeostasis.

### 2.2. The Effects of Hibernation on the Structure of the Host’s Gut Microbiota

The taxonomic changes in the microbiota of 13-lined ground squirrels were closely linked to the substrate preferences of important species in the mammalian gut microbiota [24]. Hibernation is associated with the decline in the abundance of specific taxa, like Lachnospiracea, that typically thrive on dietary plant glycans. Conversely, it leads to a rise in the proportion of taxa that excel in breaking down host-derived substrates, like *A. muciniphila* [6,25]. Certain Bacteroides, exhibiting metabolic adaptability, can dynamically shift their foraging preferences between dietary and host-derived substrates based on availability, and also exhibit an increase in abundance. The breakdown of mucin by bacteria such as *A. muciniphila* leads to the liberation of sulfates. This process might be a plausible explanation for the observed elevation in the increase in the abundance of sulfur-reducing *Desulfovibrio* in hibernating 13-lined and Arctic ground squirrels when contrasted with its level in the summer [20,26]. A study about Daurian ground squirrels revealed notable alterations in the structure and capacity for functions within the gut microbiota during hibernation. Specifically, the relative abundance of the phylum Bacteroidetes increased, while the phyla Firmicutes and Verrucomicrobia decreased in relative abundance in the Daurian ground squirrel gut microbiota [16]. PICRUSt analysis of gut microbiota showed that, during hibernation, the significant enrichment of pathways associated with carbohydrate metabolism and pentose phosphate occurred. In contrast, the control group exhibited enrichment in pathways linked to lipid biosynthesis and isoprenoid biosynthesis [16]. These results suggested a metabolic shift within the gut microbiota of Daurian ground squirrels in hibernation, favoring the breakdown of polysaccharides [27] and carbohydrates, ensuring an adequate energy supply for the host, and supporting their survival during the hibernation period [16,28]. The 16S rRNA sequencing analysis of brown bear feces across different seasons, along with unweighted UniFrac analysis, revealed distinct patterns in the gut microbiota composition [17]. As winter sets in, compared with summer, the relative abundance of Bacteroidetes has a notable surge, while the abundance of Actinobacteria and Firmicutes diminishes. These shifts in the makeup of the brown bear’s gut microbial community during winter are primarily attributed to the absence of nutritional resources during the hibernation period. The ability of Bacteroidetes to adapt to host-derived polysaccharides in the absence of external substrates, combined with the substrate preferences of Firmicutes, contributes to the discernible alterations in the gut microbiota structure during the winter months [17]. Furthermore, the greater horseshoe bat serves as a small mammal model for studying hibernation-related mechanisms. In a study utilizing 16S rRNA amplicon sequencing and PICRUSt to predict the diversity, composition, and function of gut microbiota at different periods, results indicated that, during late summer, there was notably greater diversity observed in the gut microbiota compared to the winter season [18]. During hibernation, there was a significant reduction in the proportion of Proteobacteria and Firmicutes, while Bacteroidetes showed a significant increase. In the gut microbiota of the greater horseshoe bat, Proteobacteria took center stage, accompanied by relatively lower proportions of Bacteroidetes and Firmicutes [18]. Conversely, in the gut microbiota of other mammals like ground squirrels and brown bears, Bacteroidetes and Firmicutes were the major players, while Proteobacteria made up only a small fraction. Furthermore, there was no occurrence of an elevation in Verrucomicrobia, including *Akkermansia muciniphila*, during the hibernation phase in brown bears, which is different from small mammalian hibernators. While the primary gut microbiota varied among bats and certain other mammals, there appeared to be commonalities in the transformations of their gut microbiota during hibernation [18].

During hibernation, the dominant degraders (the predominant microbial communities) change, and the sub-degraders that utilize the enzymatic activities of the dominant degraders for metabolic activities (cross-feeding) [29] also experience alterations. For example, in hibernating ground squirrels, there was a sharp decrease in the abundance of *Lachnospiracea* [26], which prefers metabolizing plant polysaccharides [30]. Consequently, this results in a decrease in the abundance of *Lactobacillus* (a sub-degrader of lactose and plant monosaccharides). The fundamental reason for this is the insufficient substrate supply during the winter, which is inadequate to sustain their metabolic activities. The combined findings from the mentioned studies revealed significant alterations in the gut microbial composition among various species throughout hibernation [16,17,18,21]. A common trend observed was the decrease in the abundance of numerous taxa compared to the summer, with an increase observed only in microbial communities capable of adapting to environmental impacts. This shift is primarily attributed to the scarcity of dietary substrates during winter. Furthermore, the enrichment of specific metabolic pathways during hibernation reflects the adjustment of energy and metabolic processes in mammals throughout this period [31]. In summary, changes in the configuration of the gut microbiota induced by hibernation may contribute to maintaining homeostasis through interactions with the host.

### 2.3. The Effects of Hibernation on the Metabolites of the Host’s Gut Microbiota

The byproducts produced by the gut microbiota, particularly short-chain fatty acids (SCFAs) that provide energy and modulate the immune system, play a vital role in maintaining the homeostasis of the host’s nutrient absorption, metabolism, immune system [32], and even nervous system [33]. During hibernation, changes occur in the diversity and composition of the host’s gut microbiota, resulting in modifications to their metabolites [34], and further causing a series of adaptive changes in the host. The targeted metabolite analysis of the cecal contents of Arctic ground squirrels was conducted using gas chromatography–mass spectrometry (GC-MS). This research revealed seasonal changes in the concentrations of SCFAs in the intestine [26]. The results suggested a noteworthy reduction in the total SCFA concentration in the cecum of ground squirrels during winter, as opposed to its summer levels. This decrease is presumably attributed to the scarcity of substrates necessary for bacterial fermentation during the winter fasting period [26]. Taking into account the periodic arousals during hibernation in Arctic ground squirrels, it was observed that the overall levels of SCFAs were lowest during the torpid phase of hibernation, while during the arousal periods, there was an increase in SCFAs levels [26]. This phenomenon is hypothesized to be linked to the increase in body temperature during arousal phases. The elevation in temperature is believed to contribute to heightened bacterial enzyme activity and an upregulation of bacterial metabolism, which makes the level of SCFAs higher than that during torpor [35]. However, unlike these small hibernators, during hibernation, brown bears can sustain a body temperature of around 30 °C [36], excluding the possible influence of reduced body temperature on shaping their microbiota. Therefore, this phenomenon would not occur in brown bears. Further analysis of the experimental data uncovers changes in the mole ratios of some SCFAs during the hibernation period. In summer, acetic acid ranks highest in mole ratio, succeeded by butyric acid, with propionic acid registering the lowest concentration [26]. In the cecal metabolome, this changing trend can be linked to alterations in the abundance of mucin-degrading bacteria during the hibernation period. As mentioned earlier, the proportionate prevalence of *A. muciniphila* rises throughout the hibernation, while the *Lachnospiraceae* shows a significant decrease (the initial ones produce both acetic acid and propionic acid [37], while the latter ones predominantly consist of butyric acid producers [38]). Besides SCFAs, metabolites like tryptophan (Trp) and bile acids (BA) also play a role in influencing the metabolism of immune cells, contributing to immune suppression and the modulation of inflammatory responses [39].

The metabolites generated by the gut microbiota significantly impact the health of the host. These metabolites can potentially affect various organ systems associated with hibernation, including the intestines, the liver, white and brown adipose tissues, and the brain [40,41]. Therefore, further research into the changes in the metabolites of gut microbiota during hibernation is essential for understanding other organs in hibernating animals.

## 3. Effects of Gut Microbiota on the Host during Hibernation

### 3.1. Regulating the Host’s Glucose and Lipid Metabolism

The metabolic function of gut microbiota was the most important, a fact substantiated by the comprehensive validation found in the results of functional enrichment [42]. The gut microbiota assumes a pivotal role in maintaining the host’s glucose and lipid metabolism balance. This is primarily attributed to the capacity of the gut microbiota to generate SCFAs, influencing glucose metabolism, lipid metabolism, insulin resistance, and more [43,44,45]. Therefore, a disruption in the equilibrium of gut microbiota could be linked to the onset and progression of metabolic disorders such as diabetes and obesity [46]. During hibernation, the gut microbiota undergoes a unique state. The main characteristic of hibernating mammals is the storage of fat and an increase in body weight. However, an overabundance of fat accumulation has the potential to result in liver impairment, manifesting as conditions like non-alcoholic fatty liver disease (NAFLD) [47,48]. Findings from Bao et al. indicated that Himalayan marmots in the pre-hibernation active phase exhibit a preference for dietary options abundant in unsaturated fatty acids (UFAs) [2], and there was a notable positive correlation between the intake of unsaturated fatty acids and body weight [49]. This preference is potentially attributed to the absence of desaturase enzymes in mammals, which hinders the endogenous synthesis of UFAs. UFAs are likely instrumental in facilitating the fat accumulation observed in Himalayan marmots [2]. Metagenomic analysis reveals a significant upregulation in the biosynthesis of UFAs within the *Firmicutes CAG:110*. This suggested that this specific bacterial group was actively involved in synthesizing UFAs, thereby supporting the host’s metabolism during the hibernation period. In the subsequent fecal transplantation experiments, the recipient mice demonstrated an elevation in body weight. The examination of liver tissue slices disclosed a discernible trend toward the progression or advancement of NAFLD [2]. These findings offered supplementary proof supporting the influence of the upregulation of *Firmicutes CAG:110* on the host’s lipid metabolism [2]. Using metagenomic analysis and the KEGG database, during the hibernation period, researchers identified and characterized 17 pathways that demonstrated a high enrichment in carbohydrate and lipid metabolism. In contrast to the fattening period, there was a noticeable increase in the upregulation of butyrate metabolism and fatty acid biosynthesis during hibernation. The findings suggested that *Bacteroides uniformis* plays a role in the synthesis of fatty acids and secreting butyrate before hibernation. Transcriptome analysis was conducted to compare the differential expression of genes (DEGs) in the liver between the fattening period and the pre-hibernation period. The findings revealed the significant enrichment of these DEGs in breaking down unsaturated fatty acids and the IGFBP complex. Therefore, it can be inferred that the gut microbiota and insulin regulate the host’s lipid and glucose metabolism [50,51], thus protecting the liver from damage caused by fat accumulation.

In the research concerning the gut microbiota of the greater horseshoe bats across different seasons, results from PICRUSt functional predictions and KEGG pathway analyses revealed significant changes. The prevalence of pathways related to lipid metabolism showed a notable rise during the hibernation period, while pathways related to carbohydrate metabolism significantly decreased compared to those in the summer [18]. The metabolic characteristics of the gut microbiota underwent a transition during hibernation, shifting from categories focused on carbohydrate metabolism to those on lipid metabolism [18]. This shift is likely correlated with alterations in the available substrate and corresponding changes in the composition of the gut microbiota. Furthermore, transcriptomic studies indicated DEGs are involved in metabolism during hibernation. Genes responsible for carbohydrate breakdown exhibited downregulation, while those involved in lipid breakdown showed an upregulation [18]. This trend is consistent with previous research findings, offering additional understanding regarding the metabolic adjustments in the gut microbiota of mammals undergoing hibernation [12,17].

### 3.2. Mediating the Host’s Nitrogen Cycling

Nitrogen participates in the process of synthesizing numerous nitrogen-containing large molecules, such as proteins, making it necessary to maintain the balance of nitrogen metabolism in organisms. However, during the fasting period of hibernation, it is restricted to obtaining nitrogen from dietary substrates. Some studies on ground squirrels have found that the gut microbiota of hibernators can mediate urea nitrogen recycling [10,52,53], converting urea nitrogen into metabolites that can be absorbed. These nitrogen compounds eventually re-enter the host’s protein pool [10]. Regan et al., by intraperitoneally injecting 13C, 15N-urea into 13-lined ground squirrels in different periods and observing the key steps of urea nitrogen recycling [10], discovered that a portion of unexcreted urea, not processed by the kidneys, was conveyed to the intestinal space via the urea transporter protein (UT-B) [54]. In the presence of gut microbiota, urea was hydrolyzed into NH3 and carbon dioxide. Furthermore, in winter, the concentration of urea in the plasma and the level of NH3 in the intestinal lumen of ground squirrels were both significantly lower than those in the summer [10]. In the subsequent stable isotope respiratory analyses, by comparing with antibiotic-treated groups, the urea decomposition activity of gut microbiota was further demonstrated. During this process, some of the generated carbon dioxide was absorbed into the bloodstream and exhaled through respiration, while NH3 was either utilized by the gut microbiota or absorbed by the host organism. The final results indicated that, during hibernation, urea nitrogen recycling was mediated by the gut microbiota, and the nitrogen is ultimately recycled and utilized in physiological activities within the cecum, muscles, and liver [10]. This research conclusively demonstrated the role of the gut microbiota in protein synthesis during hibernation. In another study on Arctic ground squirrels, it was found that during hibernation, the mucosa of the small intestine plays a pivotal role as a significant location for the pronounced expression of gut microbial urease and amino acid (AA) metabolic genes [55]. Additionally, the overall structure and function of the microbiota are different from other sections of the gut during this period. Also, six bacterial genera were found that they are responsible for 99% of urease gene expression [55].

The gut microbiota-mediated urea nitrogen recycling is a mechanism to enhance protein synthesis under extreme conditions. This holds profound significance. Conducting further research on the fundamental mechanisms of this phenomenon has the potential to advance the development of innovative therapeutic strategies for conditions such as muscle atrophy and other related diseases of humans.

### 3.3. Remodeling the Host’s Intestinal Immune System

The bacterial fermentation products derived from the gut microbiota, especially SCFAs, are crucial factors in stimulating and regulating both the immune system in the host’s intestines and the intestinal barrier function [56,57]. The immune system in the intestines functions as the key detector, identifying changes in the gut microbiota [58], driven by the scarcity of dietary substrates during hibernation and the prompt fluctuations in short-chain fatty acid levels [59]. These changes culminate in a remodeling of the host’s intestinal immune system throughout the hibernation period. In the research conducted on the 13-lined ground squirrel, it was observed that in the state of hibernation, the overall abundance of the gut microbiota plummeted roughly tenfold in comparison to that in the summer. This decrease translated to lower levels of metabolites from the gut microbiota, including SCFAs [12]. Due to the reduction in butyrate levels, the growth of intestinal epithelial cells was suppressed, consequently impacting the repair and maintenance of the intestinal barrier [58,60]. Because of the lack of dietary substrates in hibernation, mucin glycans become the main source of microbial metabolism. This results in an elevated relative abundance of Akkermansia, known for focusing on breaking down and utilizing the mucus produced by the host and its role as a producer of butyrate [61], and this can be one possible reason for the stability of the host’s intestinal immune system. Subsequent research has indeed supported the hypothesis that hibernation can stimulate host metabolic homeostasis and the expression of immune-related pathways [62,63]. The correlation between the expression of Toll-like receptors (TLRs) in the epithelial cells of the cecum and the variations in mucosa-associated microbiota is evident [64]. In hibernating 13-lined ground squirrels, a decrease in TLR4 expression, alongside an elevation in TLR5 expression, was observed. TLR4 signaling is pro-inflammatory, while TLR5 signaling is recognized for its anti-inflammatory properties [65], suggesting that this might be a protective alteration for the host’s immune function. Moreover, a significant rise in IgA + B lymphocytes was noted within the epithelium and lamina propria of the small intestine in ground squirrels. IgA, present in the mucus layer, exhibits a protective function by preventing bacteria from adhering to the epithelial cells of the intestines [66]. And in hibernation, there was a significant rise in the anti-inflammatory cytokine interleukin (IL)-10 [12]. In scrutiny of Daurian ground squirrels, the findings indicated that during hibernation, there was a reduction in the thickness of the colonic mucosa and crypt depth, a decrease in the number of goblet cells (GCs), and structural damage to certain microvilli [23]. This phenomenon may also be influenced by changes in the levels of gut microbiota metabolites (mainly SCFAs and BA). Furthermore, during hibernation, alterations in the microbiota resulted in changes to metabolites such as tryptophan and bile acids, which can influence the metabolism of immune cells, subsequently impacting immune function [39].

**Figure 1:**
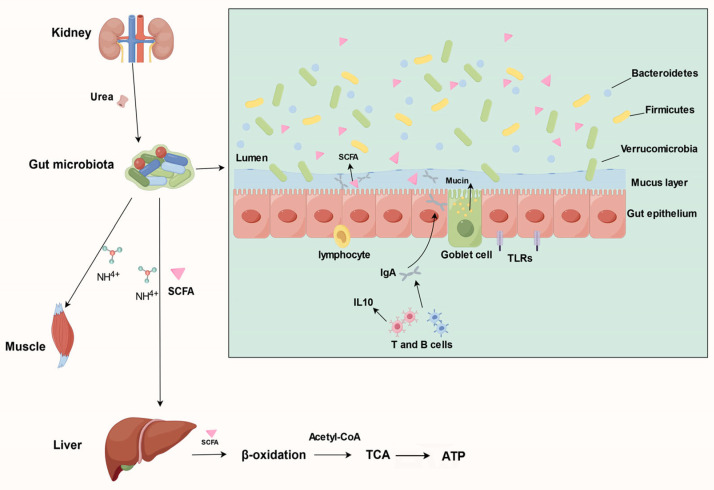
Some interactions between gut microbiota and host during hibernation. Starting from the kidneys, nitrogen cycling is mediated by the gut microbiota (**left**). Glucose and lipid metabolism are mediated by the liver and involve the metabolites of the gut microbiota, especially SCFAs (**left**). The gut immune system is regulated by changes in the gut microbiota and its metabolites (**right**).

As for how the gut microbiota influences the host’s immune function, metabolites may serve as essential bridges in the reciprocal interaction between the microbiota and the host [67]. Therefore, they represent a key entry point for investigating the specific mechanisms underlying the interplay between the immune system and the microbiota. Figure 1 shows a general overview of the impact of gut microbiota changes during hibernation on host metabolism, including the urea nitrogen cycle, immune regulation processes, and lipid metabolism processes.

## 4. The Effect of External Factors on the Host’s Gut Microbiota

### 4.1. The Effect of Dietary on the Host’s Gut Microbiota

Diet is not only crucial for sustaining body growth, reproduction, and overall health, but also it influences and provides support for the symbiotic microbial communities residing in the digestive tract—referred to as the gut microbiota [5]. In the study of pre-hibernation fattened Arctic ground squirrels, three kinds of diets with varying fat content were employed [68]. After five weeks, the researchers compared the diversity and functionality of the gut microbiota in squirrels. The results indicated that the fattened time did not exhibit notable distinctions among the three groups of squirrels. However, distinct functional differences were observed in the gut microbiota of the squirrels subjected to different diets [68]. The research indicates that the diet before hibernation affects the function of Arctic ground squirrel’s gut microbiota. However, there is not a distinctly evident correlation observed between the diet and the structural composition of the gut microbiota. Research on common hamsters has indicated that the fat content in the diet before hibernation can influence the torpor period [69,70]. Specifically, hamsters on a high-fat diet before hibernation experience more weight gain and less torpor time in comparison to individuals following a high-protein diet, and they lose more weight during the hibernation period [70]. While the dietary structure before hibernation may not directly correlate with the gut microbiota during hibernation, the dietary form and structure can affect the host’s physical characteristics, especially body weight [2]. This inevitably raises the question of whether dietary effects will cause microbial changes and whether they may play a role in the overall adaptation of the microbiota to hibernation. Further experiments are required to elucidate the detailed mechanisms through which pre-hibernation diet impacts the hibernation process.

The dietary habits of mammalian hibernators, including fasting levels, play a crucial role in shaping the intricate dynamics of their physiological processes. For instance, 13-lined ground squirrels refrain from eating throughout their entire hibernation, whether they are awake or not [20]. Similarly, male Arctic ground squirrels, despite storing food before hibernation, abstain from eating during the hibernation period. Instead, they consume the stored food just several days before emergence from hibernation [71]. Syrian hamsters, categorized as food-caching hibernators, depend on stored food to meet their energy needs during the hibernation period [72]. Considering the variable fasting levels experienced by different hibernators during hibernation, this results in adaptive alterations in the gut microbiota to varying extents. The structure of the diet and the manner of feeding impact both the diversity and the function of the host’s gut microbiota. Therefore, studying the dietary impact on mammalian hibernation can enhance our comprehension of this physiological process. It may also aid in the development of novel biomedical strategies.

### 4.2. The Effect of Temperature on the Host’s Gut Microbiota

In a study on the greater horseshoe bat [73], two different caves were identified as roosting sites, with identical temperatures during the summer but differing by 5 °C in the winter. Using the high-throughput sequencing of the 16S rRNA gene, the diversity and composition of the bat gut microbiota were analyzed during both summer and winter in the two caves. The results indicated that the gut microbiota of the heterothermic animal, the greater horseshoe bat, was influenced by temperature variations. Bats hibernating at lower temperatures exhibited more pronounced changes in the structure of their gut microbial community, thereby impacting energy-related metabolic pathways [73]. However, in another study, subjecting Arctic ground squirrels to temperature elevation during hibernation while maintaining fasting conditions did not result in changes in the concentration and proportion of SCFAs in the cecum [26]. This suggests that, in hibernating animals, the scarcity of food may have a more pronounced influence on the metabolic byproducts of microbial activity than temperature. However, due to the lack of measurement of changes in the diversity and structure of the gut microbiota during this process, at present, the available evidence is insufficient to affirm that temperature variations do not influence the gut microbial community.

The current global warming trend is alarming, and the structure and function of gut microbiota are highly sensitive to environmental variables. Further research on the influence of temperature on the gut microbial community in hibernating animals contributes to the understanding of the effects of the global warming scenario on animals and aids in the development of mitigation measures [74].

### 4.3. The Effect of Photoperiod on the Host’s Gut Microbiota

The photoperiod serves as a crucial signal for physiological processes in animals [75]. Research indicated that the photoperiod influences the circadian clock, as well as rhythmic behaviors such as feeding and activity. Furthermore, the light–dark cycle can also trigger hibernation by influencing the secretion of melatonin [76]. Recent research has proposed that there may be interactions between the photoperiod and the gut microbiota in animals [77,78,79], which may be linked to hibernation. Through altering photoperiod and performing fecal transplant experiments, notable variations in the gut microbiota of male Brandt’s voles have been identified under different light–dark cycles. Certain microbes were notably associated with hormones like melatonin and genes such as hypothalamic Kiss-1 and testicular Dio-3. When these gut microbes were transplanted into recipient voles, their circadian rhythms were also notably affected [78]. Rats induced into obesity through diet were subjected to three different light cycle treatments over nine weeks. During this period, when these rats were fed with grape seed proanthocyanidins (GSPEs) alone or a combination of GSPEs and antibiotics, those under a 6 h light cycle demonstrated the increased bioavailability of GSPE in comparison to that of the 18 h light cycle group. However, rats treated with antibiotics nullified these photoperiod-mediated changes [77].

To sum up, there seem to be some specific interactions between the photoperiod and animals, particularly concerning the composition of the gut microbiota. The gut microbiota is responsive to changes in the photoperiod, playing a regulatory role in shaping the circadian rhythms of the host.

## 5. Conclusions

During hibernation, mammals undergo significant changes in their gut microbiota in response to extreme environmental shifts. This alteration in the microbiota, in turn, influences the host, establishing a dynamic equilibrium throughout hibernation. This reciprocal interaction sheds light on how hibernating mammals overcome challenges associated with this state. The scarcity of dietary substrates and fasting during hibernation impact the diversity, structure, and metabolite levels in the intestinal microbiota. Research tracking these changes reveals adaptive alterations, particularly in pathways related to metabolism and immune regulation. Collectively, these adaptations contribute to the host’s stability during hibernation. Studying the interplay between gut microbiota and hibernating hosts enhances our understanding of adaptability and survival in extreme environments, offering potential insights for medical applications, especially in addressing metabolic and immune-related diseases. Moreover, exploring hibernating mammals from a biological perspective may uncover evolutionary changes during prolonged survival, with the gut microbiota–host interplay being a pivotal factor in these adaptations.

## 6. Future Research Directions

Research on hibernating mammals has shown that hibernation has a relative plasticity. The specific issue that needs to be thoroughly addressed is how the gut microbiota of hibernators participates in shaping homeostasis over a while through a series of complex interactions with environmental changes. The extreme environmental conditions experienced by hibernators, if translated into humans, are equivalent to pathological states, such as NAFLD and nitrogen cycling homeostasis mentioned in the article. If this self-regulation mechanism can be decoded and applied to humans, it may enhance their adaptability to external factors, such as the environment of human spaceflight environment, and provide more effective treatment methods for human diseases. Therefore, the study of the mechanisms by which hibernating mammals maintain their homeostasis has broad application prospects.

## Data Availability

Not applicable.

## Abbreviations

OTUs	Operational Taxonomic Units
SCFAs	Short-chain fatty acids
GC-MS	Gas chromatography–mass spectrometry
Trp	Tryptophan
BA	Bile acids
NAFLD	Non-alcoholic fatty liver disease
UFAs	Unsaturated fatty acids
DEGs	Differential expression of genes
UT-B	Urea transporter protein
TLR	Toll-like receptor
GSPEs	Grape seed proanthocyanidins
